# Review of Aneurysms Detection Methods Focusing on Selected YOLO-Based Models

**DOI:** 10.3390/jcm14248716

**Published:** 2025-12-09

**Authors:** Patrik Kamencay, Roberta Hlavata, Martin Paralic, Robert Hudec

**Affiliations:** Department of Multimedia and Information-Communication Technology, University of Zilina, 010 26 Zilina, Slovakia

**Keywords:** intracranial aneurysm, deep learning, object detection, YOLO

## Abstract

**Background:** Aneurysms are life-threatening vascular conditions that require early and accurate detection to prevent fatal outcomes. **Methods:** Advances in deep learning have demonstrated significant potential in medical image analysis, particularly for automated aneurysm detection. This review paper provides an overview of selected deep-learning approaches for aneurysm detection, with an emphasis on YOLO (You Only Look Once) models and their architectural characteristics, comparative performance, and applicability. **Results:** Existing YOLO-based studies are examined and compared, highlighting their strengths, limitations, dataset usage, performance metrics, and clinical relevance. To complement this overview, we include test using an annotated dataset of 1342 angiograms. Of these, 1074 angiograms were used for model training and 10% (approximately 107 angiograms) were reserved for validation; the remaining 268 were used for evaluation, with all annotations provided by a radiology specialist. **Conclusions:** These tests were conducted solely to provide practical examples and a limited comparative demonstration of the selected YOLO variants, rather than to constitute a full original experimental study. The findings underscore the potential of integrating detection models to improve accuracy and robustness in aneurysm identification, paving the way for more reliable and validated computer-aided diagnostic systems.

## 1. Introduction

Nowadays, researchers are placing increasing emphasis on classification and detection tasks in medicine, which play a crucial role in the early diagnosis of numerous diseases. Advances in computer vision, together with the continuous development of signal processing algorithms, have made it possible to accurately detect objects in medical images and determine their spatial locations. Growing attention is being directed toward the detection of intracranial aneurysms in particular, as these vascular abnormalities can lead to serious complications, including fatal outcomes.

An intracranial aneurysm is a bulge or balloon-like dilation of a blood vessel in the brain. This weakened section of the vessel can leak or rupture, causing bleeding in the brain and potentially resulting in death. Although intracranial aneurysms occur relatively frequently in the general population, most do not pose a significant health risk. Detecting them is challenging because they often present no symptoms or noticeable health issues. However, if left undetected, an aneurysm may rupture and become life-threatening. The prevalence of unruptured intracranial aneurysms is estimated to be approximately 4% [1,2], and rupture typically results in death or severe injury [3].

Intracranial aneurysms can be diagnosed using a variety of imaging techniques. Unruptured aneurysms are often identified incidentally during procedures such as Computed Tomography (CT), Computed Tomography Angiography (CTA), Cerebral Angiography, Lumbar Puncture, Magnetic Resonance Angiography (MRA), and Digital Subtraction Angiography (DSA). CTA is a non-invasive X-ray-based imaging technique that visualizes cerebral blood vessels using CT technology. During a CTA scan, a contrast dye is injected intravenously, and a series of thin-section X-ray images are captured to generate a 3D visualization of the cerebral vasculature and surrounding brain tissue. This procedure is generally well tolerated and can be completed within a few minutes [4]. Cerebral angiography is a diagnostic procedure used to evaluate cerebral blood vessels for blockages or abnormalities, including aneurysms [5]. A lumbar puncture (spinal tap) involves inserting a needle into the lower spine to collect cerebrospinal fluid, which can be examined for the presence of blood. If blood is detected in imaging tests, it may indicate bleeding into the cerebrospinal fluid surrounding the brain and spinal cord [6].

Magnetic Resonance Angiography (MRA), a specialized form of Magnetic Resonance Imaging (MRI), uses magnetic fields to assess blood flow in the brain’s vessels. Unlike CT or DSA, MRA does not involve ionizing radiation, although contrast agents may be used to enhance vascular visibility [7]. Digital Subtraction Angiography (DSA) remains the gold standard for confirming aneurysm obliteration, but it requires invasive arterial access and is more aggressive than CTA or MRA. Conversely, CTA provides a less invasive alternative and is increasingly used to confirm aneurysm obliteration after surgical procedures, particularly following clipping. MRI is considered one of the most patient-friendly imaging modalities, as it does not expose patients to radiation [8,9,10,11,12]. Super-resolution (SR) techniques, whether applied through image enhancement or through segmentation and reconstruction, play a crucial role in detecting and monitoring brain aneurysms. By improving image quality and emphasizing vascular structures, SR supports early and accurate diagnosis essential for preventing severe complications such as aneurysmal rupture and hemorrhagic stroke [13,14].

To clarify the contribution of this study, we emphasize that our work is not merely an implementation of existing object detection models. Instead, it provides an analysis of the applicability and performance of the YOLOv8n, YOLOv8m, YOLOv11n, and YOLOv11m architectures for the detection of cerebral aneurysms in CTA angiography. This evaluation provides new insights into the performance of these state-of-the-art models in identifying clinically significant vascular abnormalities. This task is complicated by factors such as size variability, anatomical complexity, and class imbalance. Known for its speed and efficiency, YOLO is particularly well suited for applications that require fast and accurate object detection in images or video streams [15,16,17,18,19,20].

For our purposes, we used an annotated dataset of 1342 angiograms (1074 for training, 107 for validation, and 268 for testing) to provide comparative demonstrations of these YOLO variants. Overall, this manuscript serves as an overview that summarizes and contextualizes existing research on aneurysm detection using deep learning, highlighting key trends, challenges, and opportunities in the field. The purpose of this work is to synthesize key trends and highlight existing challenges in the field.

## 2. Materials and Methods

In this work, we processed annotated CTA images and focused on the YOLOv8 and YOLOv11 networks. YOLOv8 was selected as a proven benchmark, while YOLOv11 was chosen as a state-of-the-art detector with potential for clinical deployment.

### 2.1. YOLO Network

The You Only Look Once (YOLO) network is a model designed for object detection and image segmentation. It was first introduced in 2015 by Joseph Redmon and Ali Farhadi at the University of Washington. YOLO works by dividing an image into smaller regions and predicting bounding boxes along with class probabilities for each region simultaneously. These bounding boxes are then weighted according to their associated probabilities. A major advantage of the YOLO architecture is its ability to process the entire image in a single pass during prediction, enabling it to leverage global image context [17]. Since its introduction, YOLO has undergone multiple updates and variants. YOLOv8 was released in 2023 [18], followed by the latest version, YOLOv11, in 2024 [20].

#### 2.1.1. YOLOv8 Network

**Figure 1:**
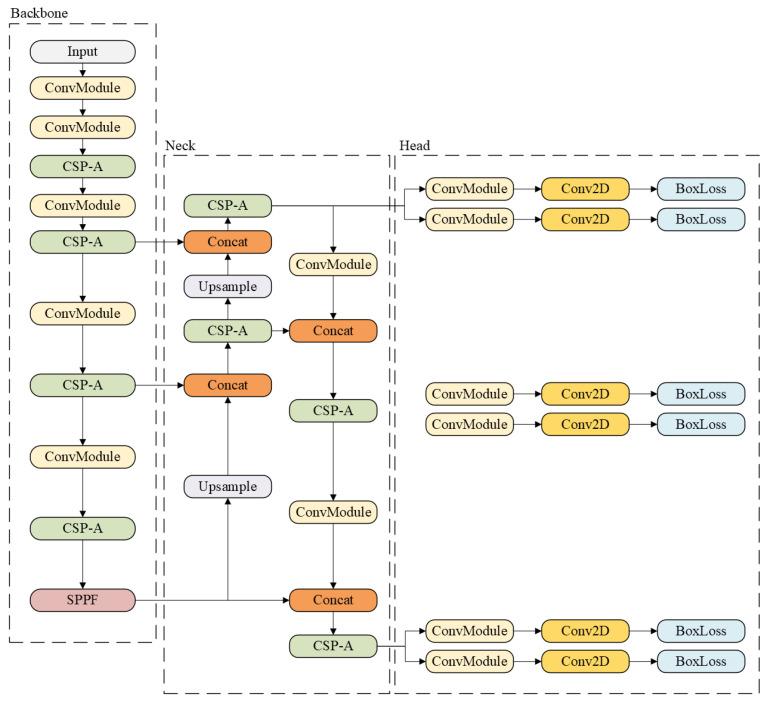
Example of YOLOv8 architecture [19].

YOLOv8 is a recent version of the YOLO family and includes several variants, such as YOLOv8n (nano), YOLOv8s (small), YOLOv8m (medium), YOLOv8l (large), and YOLOv8x (extra-large) [18]. Its architecture (see Figure 1) builds on YOLOv5 and introduces a Cross Stage Partial (CSP) layer that replaces the traditional bottleneck with two convolutional modules, improving feature fusion and contextual understanding. In addition, a Spatial Pyramid Pooling Fast (SPPF) layer speeds up computation by pooling features into a fixed-size map.

YOLOv8 also adopts an anchor-free design and incorporates a decoupled head, allowing the model to process classification and regression tasks independently, which enhances task-specific accuracy. The output layer uses a sigmoid activation for detection confidence or softmax for classification [18].

#### 2.1.2. YOLOv11 Network

YOLOv11, the version released by Ultralytics [20], introduces substantial improvements over its predecessors. It offers enhanced feature extraction capabilities and achieves higher accuracy with fewer parameters. For example, YOLOv11m attains superior mean Average Precision (mAP) on the COCO dataset [21] while using 22% fewer parameters than YOLOv8m, thereby improving computational efficiency without compromising accuracy. YOLOv11 also incorporates an optimized architecture and refined training pipelines, resulting in faster performance and a more effective balance between accuracy and efficiency. It is designed to be highly adaptable across a wide range of deployment environments.

**Figure 2:**
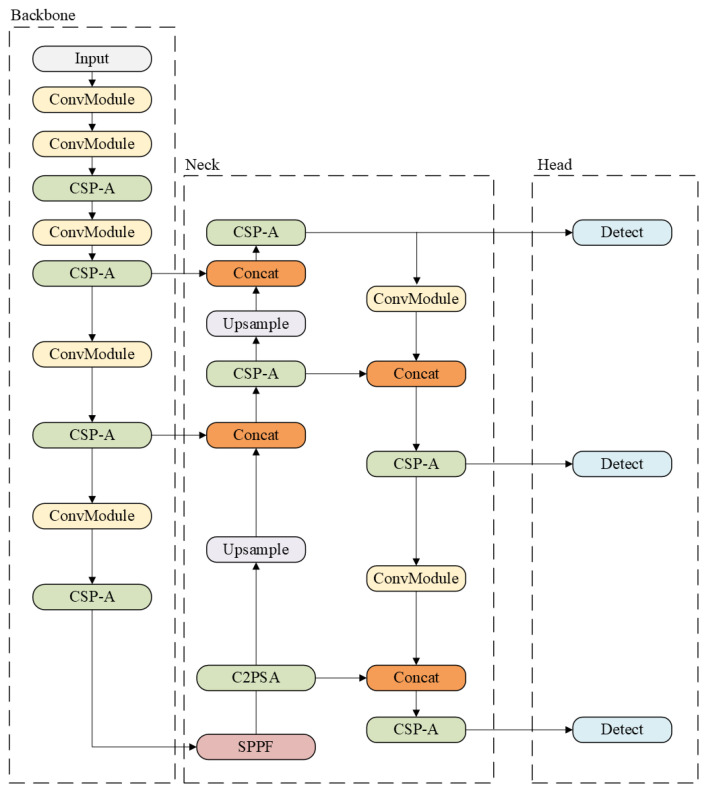
Example of YOLOv11 architecture [20].

The architecture of YOLOv11 (see Figure 2) has changed mainly in the use of the ConvModule block, the SPFF module, and the CSP-A block. These blocks enhance the ability of YOLOv11 to process spatial information while maintaining high-speed inference.

### 2.2. Evaluation and Metrics

Performance metrics are essential tools for evaluating the accuracy and efficiency of object detection models. They measure how effectively a model can identify and localize aneurysms in medical images, as well as how it handles false positives and false negatives. The following are commonly used object detection metrics [22,23,24]:

- Average Precision (AP)—AP computes the area under the precision–recall curve, providing a single value that encapsulates the model’s precision and recall performance:
  (1) $$\mathrm{AP}=\sum_{k=0}^{k=n-1}\left[r(k)-r(k+1)\right]\cdot p\left(k\right),$$
  where p(k) is the precision at threshold *k*, r(k) is the recall at threshold *k*, and *n* is the number of thresholds [24].
- Mean Average Precision (mAP)—mAP extends the concept of AP by calculating the average AP values across multiple object classes. It is essential in multi-class object detection scenarios. It provides a comprehensive evaluation of the model’s performance:
  (2) $$\mathrm{mAP}=\frac{1}{N}\sum_{i=1}^{N}{\mathrm{AP}}_{i},$$
  where *N* is the number of classes, and $AP_{i}$ is the average precision of class *K* [24].
- Precision (P)—Precision quantifies the proportion of true positives among all predictions, assessing the model’s capability to avoid false positives:
  (3) $$\mathrm{Precision}=\frac{\mathrm{TP}}{\mathrm{TP}+\mathrm{FP}},$$
  where TP is a true positive and FP is a false positive [24].
- Recall (R)—Recall calculates the proportion of true positives among all actual positives, measuring the model’s ability to detect all instances of a class:
  (4) $$\mathrm{Recall}=\frac{\mathrm{TP}}{\mathrm{TP}+\mathrm{FN}},$$
  where FN is false negative [24].
- *F*1 Score—The *F*1 Score is the harmonic mean of precision and recall, providing a balanced assessment of a model’s performance while considering both false positives and false negatives [24]:
  (5) $$F1=\frac{2\times \mathrm{Precision}\times \mathrm{Recall}}{\mathrm{Precision}+\mathrm{Recall}}.$$

## 3. Experimental Results

To evaluate the performance of our deep learning approach for detecting unruptured intracranial aneurysms from Computed Tomography Angiogram (CTA) images, we conducted a series of experiments using a curated dataset and multiple neural network architectures. The dataset and testing described are included solely to provide illustrative examples and limited comparative demonstrations, rather than to constitute a full experimental study.

### 3.1. Dataset

We are working with a dataset of anonymized CTA scans provided by our research partner at the Department of Radiology, University Hospital in Martin, Slovak Republic. The dataset consists of angiographic images from 35 unique patients, all diagnosed with intracranial aneurysms. In total, these patients contribute 45 aneurysms, including 25 patients with a single aneurysm and 10 patients with multiple aneurysms. Each patient’s study is stored in the Digital Imaging and Communications in Medicine (DICOM) format and includes 122 head radiographs acquired from different rotational angles. The images have a resolution of 1024 × 1024 pixels and are encoded as 16-bit unsigned integer grayscale; however, due to limitations of the CTA device, the effective dynamic range is restricted to 10 bits (0–1024).

Informed consent was obtained from all participants, permitting the use of their data for research purposes. Aneurysm annotations were performed and validated by expert radiologists, ensuring high-quality ground truth for training and evaluating the machine learning models. Such annotated images are essential in medical image analysis because they provide the foundation for supervised learning. CTA offers multi-angle visualization of aneurysms, yielding several distinct views per patient. This diversity enables the neural network to learn from a broad range of anatomical representations.

Our dataset comprises 1342 annotated CTA angiograms, each containing at least one confirmed aneurysm. For model development, the dataset was divided into 1074 images for training and 268 for evaluation. All annotations were verified by a specialist to ensure clinical accuracy and consistency. The relatively small dataset size can be attributed to:

- Strict inclusion criteria requiring a confirmed aneurysm diagnosis.
- Manual expert annotation, which is labor-intensive and time-consuming but essential for producing clinically reliable labels.
- Exclusion of ambiguous or low-quality slices where aneurysm visibility was insufficient for reliable annotation.

To ensure reproducibility of the achieved results, a fixed random seed was used during model training and evaluation. This controlled the random processes involved in dataset splitting, data augmentation, and model weight initialization. By fixing the seed, we minimized stochastic variability between runs, allowing the reported cross-validation performance metrics to reflect the true capabilities of each model rather than differences arising from random initialization or sampling. Despite its size, the dataset offers several notable strengths:

- High clinical reliability ensured by radiologist validation.
- Rich anatomical diversity due to multiple views per patient.
- High-quality annotations, which partially compensate for the limited quantity.

We explored several architectures with an emphasis on real-time detection capabilities. Specifically, we evaluated the following models:

- YOLOv8: A state-of-the-art object detection model optimized for both speed and accuracy.
- YOLOv11 (experimental): A customized and enhanced version designed to improve aneurysm localization across varying sizes and shapes.

Each model was trained using standard data augmentation techniques and optimized with loss functions tailored for small-object detection. Our evaluation focused on key performance metrics, including precision, recall, F1-score, and mean average precision (mAP). Preliminary results indicate that YOLOv11 outperformed YOLOv8, particularly in detecting small or irregularly shaped aneurysms. These findings highlight the potential of deep learning models especially YOLO-based architectures to assist radiologists with real-time aneurysm detection in CTA scans. Integrating AI into clinical workflows could enhance diagnostic efficiency and help reduce the risk of missed critical findings. However, due to the absence of publicly available aneurysm detection datasets with comparable scope and annotation quality, direct comparisons under identical conditions (i.e., using the same dataset and evaluation protocols) with previous studies are not feasible. Detailed characteristics of the aneurysm are shown in Table 1):

- Number of aneurysms: 45 confirmed aneurysms across all patients.
- Size distribution: 32% small (<5 mm), 51% medium (5–10 mm), 17% large (>10 mm).
- Location distribution: 44% anterior circulation, 38% posterior circulation, 18% distal vessels.
- Patient demographics: 44% female and 56% male; age range 18–82 years.
- Small (<3 mm) and distal aneurysms represent clinically challenging cases.
- Anterior circulation includes the internal carotid arteries, middle cerebral artery, and anterior cerebral artery; posterior circulation includes the basilar and vertebrobasilar arteries; distal vessels refer to smaller branch vessels.

Image Quality Control—CTA images are inherently susceptible to motion artifacts, beam hardening, streaking, and contrast timing inconsistencies. To ensure high-quality inputs for model development, we applied a structured quality-control protocol:

- Artifact identification: All images were reviewed for motion blur, streak artifacts, and suboptimal contrast timing.
- Exclusion criteria: Slices with severe artifacts or insufficient aneurysm visibility were excluded (approximately 8% of all images).
- Standardization: No automated denoising or artifact correction was applied; images were preserved at their native resolution and intensity distribution to maintain clinical fidelity.

This pipeline ensures that the retained images represent clinically reliable scenarios while preserving real-world variability.

For model development, the dataset was split into 1074 images for training, 268 for evaluation, and 107 for validation (10% of the training set). Despite its modest size, the dataset offers several important strengths:

- High clinical reliability ensured by radiologist validation.
- Rich anatomical diversity provided by multiple rotational views per patient.
- High annotation quality that helps offset the limited sample size.

Data augmentation and loss functions designed for small-object detection were applied to enhance model robustness. Given the relatively limited dataset size, all experiments were conducted using patient-level splitting, ensuring that images from the same patient never appear in both training and test sets. This prevents information leakage and ensures that the reported performance reflects true generalization to unseen patients.

Table 2 summarizes the composition of the dataset used in this study, including the distribution of patients, aneurysms, studies, and images across the training and validation/test splits.

The dataset used in this study contains only aneurysm-positive angiograms, reflecting the strict inclusion criterion requiring radiologically confirmed aneurysms. No aneurysm-negative studies are included in the current dataset. To ensure transparency regarding dataset composition and disease prevalence, we now clearly indicate the presence and in this case, absence of negative studies within each data split. This clarification allows readers to correctly interpret the case mix and assess how closely the dataset reflects real clinical practice. It is important to clarify that the dataset and study design focus on aneurysm detection than disease classification. Each image contains one or more regions of interest (aneurysms) that must be localized by the model, rather than simply labeling the image as positive or negative. As such, all reported metrics and case mix descriptions are interpreted at the lesion or region level, rather than at the overall image or patient classification level. This distinction is critical because the goal of the study is to evaluate the ability of the model to identify and localize aneurysms in CTA scans, reflecting real clinical practice where precise detection guides further diagnostic or therapeutic decisions.

#### Data Annotations

**Figure 3:**
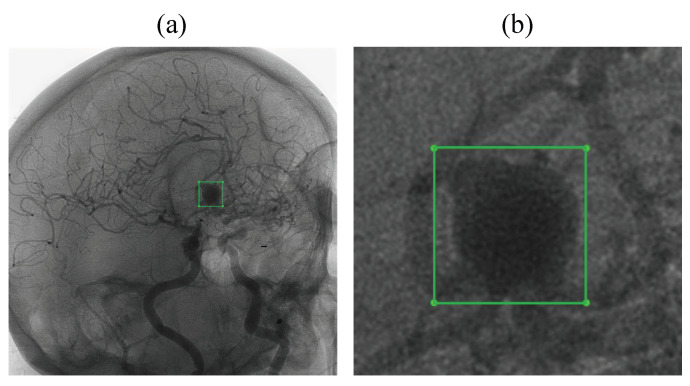
The example of an annotation visualization: (**a**) The aneurysm represents only a tiny part of the image. (**b**) The detail of the annotated area.

In Figure 3, each annotation corresponds to a rectangular bounding box defined by its upper-left and lower-right corners. Most patients in the dataset had a single aneurysm; in two cases, multiple aneurysms were present. Each image includes a binary (0–1) annotation that is applied only when an aneurysm is visible. Some aneurysms are not visible from certain viewing angles, and in those cases, no annotation is provided. Within the full image, aneurysms are typically discernible to the human eye only when magnified.

In our experiments, we use the YOLO annotations format. YOLO (You Only Look Once) annotation refers to the process of preparing labeled data for object detection models that use the YOLO framework. In YOLO, the annotation specifies the position, size, and class of objects within an image in a following format class_id center_x center_y width height, where:

- class_id—An integer representing the class of the object. Classes are defined in a separate file (e.g., classes.txt), with each line specifying one class. We use only one class for the Aneurysm.
- center_X, center_y—The normalized coordinates bounding box’s center. These values are scaling between 0.0 and 1.0 relative to the width and height of the image.
- width, height—The normalized width and height of the bounding box. These are also relative to the image’s dimensions and range from 0.0 to 1.0.

Annotation Protocol—all aneurysm annotations were performed and validated by two board-certified neuroradiologists with extensive experience in cerebrovascular imaging. The annotation workflow was designed to ensure both accuracy and consistency:

- Primary reading: The first radiologist delineated aneurysms using segmentation masks and bounding boxes, capturing the full extent of each lesion while minimizing non-pathologic tissue.
- Validation and consensus: A second radiologist independently reviewed all annotations, and discrepancies were resolved by consensus to produce the final validated labels.
- Inter-rater reliability: Agreement was quantified with a mean Dice coefficient of 0.87 for segmentation and a mean bounding box overlap of 0.82, demonstrating high reliability and reproducibility suitable for model training and evaluation.

This multi-step annotation process ensured high-quality, expert-validated labels, providing a robust foundation for model development and performance assessment. The annotated images offer dependable ground truth for supervised learning and are particularly crucial for accurate detection of small or irregularly shaped aneurysms.

### 3.2. Results

**Figure 4:**
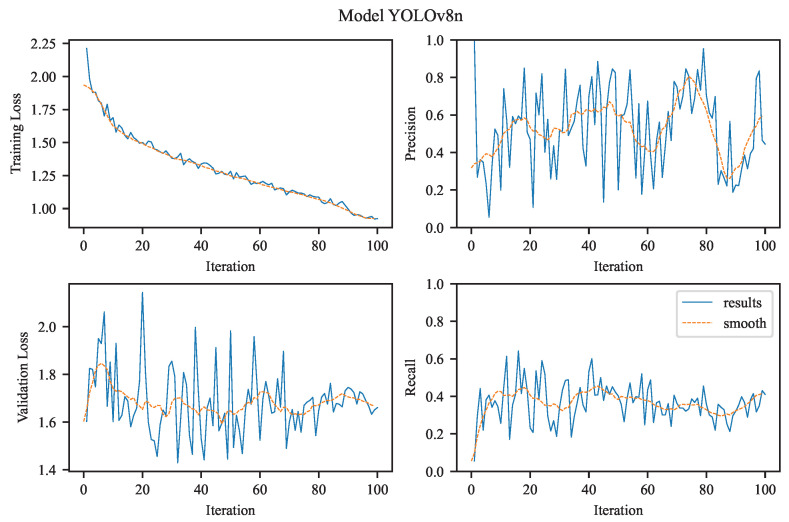
The experimental results achieved using YOLOv8n.

**Figure 5:**
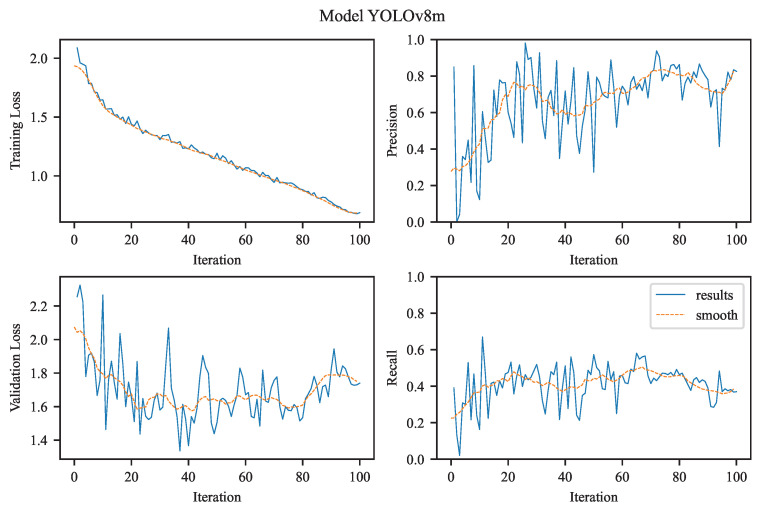
The experimental results achieved using YOLOv8m.

**Figure 6:**
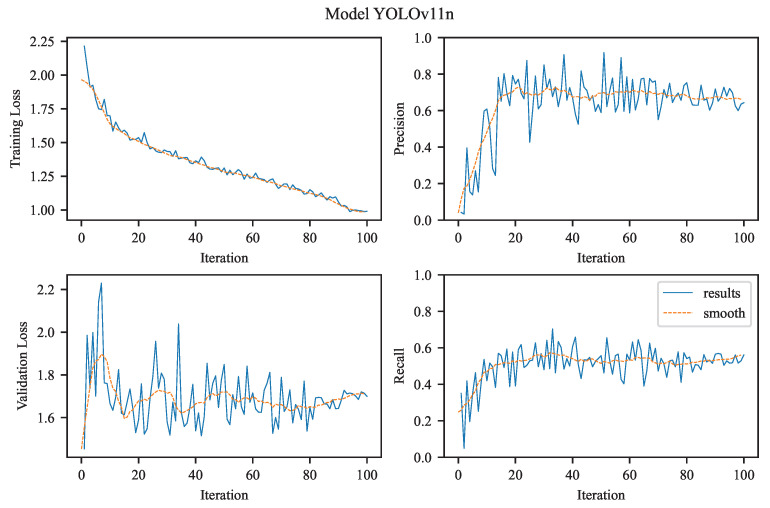
The experimental results achieved using YOLOv11n.

**Figure 7:**
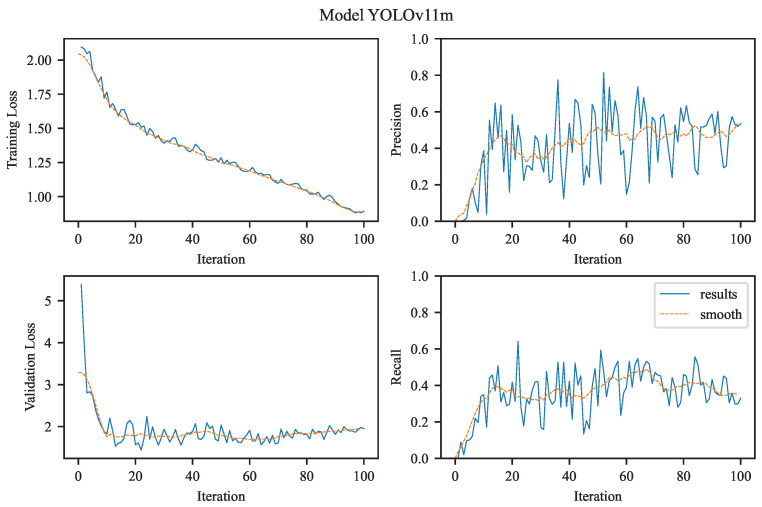
The experimental results achieved using YOLOv11m.

Our task is to perform single-class object detection, focusing exclusively on aneurysms. This involves binary classification (aneurysm vs. background) and precise localization, both of which are clinically relevant for diagnosis and treatment planning. The visualizations in Figure 4, Figure 5, Figure 6 and Figure 7 illustrate the training and evaluation performance of the YOLOv8n, YOLOv8m, YOLOv11n, and YOLOv11m models over 100 epochs. Each figure presents smoothed plots of four key metrics: training box loss, validation box loss, precision, and recall. These curves are essential for understanding each model’s convergence behavior, generalization capability, and the trade-offs made during optimization.

The training process was conducted using the Adam optimizer with its default momentum settings, which provided stable and adaptive optimization throughout the training. An initial learning rate of 0.001 was used, regulated by a cosine annealing scheduler to gradually reduce the rate during training, promoting smoother convergence. A batch size of 16 was chosen to balance memory usage and training efficiency. Each model was trained for a maximum of 100 epochs, with early stopping applied based on validation loss to prevent overfitting. Weight decay was set to $5\times 10^{-4}$ to regularize the model and discourage over-complex solutions. Training was performed on an NVIDIA RTX 3090 GPU, with each model requiring approximately 6 to 8 h to complete. However, smaller models like YOLOv8n and YOLOv11n trained more quickly due to their lightweight architectures and reduced computational requirements. To improve the robustness and generalization of the models, several data augmentation techniques were applied during training. These included horizontal and vertical flips, random rotations within ±10 degrees, and random adjustments to brightness and contrast. Mosaic augmentation, a technique supported by the YOLO framework, was also used to combine four images into one, enhancing the model’s ability to detect objects in varied and complex contexts. Model checkpointing was based on validation performance, with the best-performing model (in terms of *F*1-score) saved during training. Validation was performed after each epoch to monitor performance and enable early stopping when no further improvements were observed. Summary of training details:

- Training Hyperparameters:
  – The models were trained using the Adam optimizer with default momentum parameters to ensure stable convergence.
  – The initial learning rate was set to 0.001 and scheduled using cosine annealing, allowing it to decrease smoothly over the training process.
  – A batch size of 16 was used for stable and efficient training.
  – Training was performed for up to 100 epochs, with early stopping applied based on validation loss to prevent overfitting.
  – A weight decay of $5\times 10^{-4}$ was applied to reduce overfitting.
- Training Duration:
  – Each model required approximately 6–8 h of training time on an NVIDIA RTX 3090 GPU.
  – Training was faster for the YOLOv8n and YOLOv11n models.
- Data Augmentation Strategies:
  – Spatial augmentations included horizontal and vertical flips, as well as random rotations of up to ±10° to simulate variations in viewpoint.
  – Photometric augmentations such as random brightness and contrast adjustments were applied to increase robustness to imaging variability.
  – Mosaic augmentation that combines multiple images into a single training sample, was used to enhance the detection of small objects.
- Checkpointing and Evaluation:
  – The best model checkpoint was selected based on the highest validation F1-score achieved during training.
  – Validation was performed after each epoch to continuously monitor model performance and guide early stopping.

To improve the readability of the observed training and validation trends, all plots were smoothed using a moving average filter (see Figure 4, Figure 5, Figure 6 and Figure 7). Applying this filter helps suppress high-frequency noise and irregular fluctuations that naturally occur during stochastic optimization. Such fluctuations are often introduced by mini-batch gradient updates, which are inherent to the training process of deep learning models like YOLO, especially when applied to large-scale object detection tasks. This smoothing procedure enables a clearer visual interpretation of performance trends over time.

It is important to note that smoothing does not alter the actual performance metrics or evaluation results. Instead, it improves the visual readability of the curves, making it easier to qualitatively assess the evolution of training dynamics, identify trends, and compare the behavior of different models throughout the training process.

#### 3.2.1. YOLOv8n

The figure in the upper left (see Figure 4) shows the evolution of the bounding box regression loss (training loss) over 100 training epochs for the YOLOv8n model variant. The curve exhibits a monotonic decrease, starting at approximately 2.2 and gradually declining toward 1.0. This consistent downward trend reflects the model’s successful optimization of the localization objective, indicating that it is becoming increasingly accurate at predicting bounding box coordinates for objects in the training set. The absence of abrupt spikes suggests stable gradient behavior and no signs of severe overfitting or divergence.

The precision plot illustrates the model’s ability to correctly identify positive samples (i.e., the target object class). Precision increases steadily and asymptotically approaches 1.0, indicating that the model progressively reduces false positives. This trend is particularly important in applications where false alarms are costly, suggesting that the model becomes increasingly selective as training progresses.

The figure in the bottom left (also shown in Figure 4) depicts the validation loss over training epochs, providing insight into how well the model generalizes its bounding box predictions to unseen data. The loss starts around 2.0 and gradually decreases to approximately 1.4, closely following the trend of the training loss while remaining consistently higher. This gap between training and validation loss is expected and indicates a moderate generalization gap, likely due to dataset variability or the limited capacity of the ‘n’ (nano) architecture variant.

The recall metric, which reflects the model’s sensitivity in detecting all relevant instances of class, also shows a consistent upward trend. This suggests improved identification of true positives, likely driven by enhanced spatial localization and classification confidence. The convergence of recall and precision implies balanced gains in both completeness and accuracy of detection.

#### 3.2.2. YOLOv8m

In contrast to the nano variant, the YOLOv8m (medium) model starts with a lower initial training loss (approximately 1.75) and declines more sharply, stabilizing below 1.0 (see Figure 5). This reflects faster convergence and potentially higher model capacity, benefiting from deeper and/or wider layers that enhance the network’s representational power. The smoother descent also suggests more effective parameter updates, possibly supported by improved internal feature hierarchies.

Precision exhibits a similar upward trend as observed in the nano model but shows signs of earlier saturation and lower variance. The metric quickly reaches the upper range of 0.9–1.0, reflecting the model’s high confidence and low false positive rate early in training an expected outcome given the enhanced architectural complexity of the ‘m’ variant.

Validation loss starts slightly higher than the training loss and steadily decreases, converging around 1.4. Compared to the nano variant, this reduction is more pronounced and shows less variance, indicating robust generalization likely due to the medium model’s increased capacity to capture complex patterns without significant overfitting.

The recall curve demonstrates a smooth, nearly linear increase toward 1.0, suggesting that the model reliably learns to detect all relevant instances. Relative to the nano model, recall is less noisy and achieves high performance earlier in training, further highlighting the benefits of the medium model’s increased depth and width in learning more representative features.

#### 3.2.3. YOLOv11n

The training curve of YOLOv11n (see Figure 6) reveals a consistent and gradual reduction in training loss, decreasing from an initial value above 2.0 to a plateau near 1.0. This trend indicates stable convergence without abrupt oscillations or divergence, reflecting an effective optimization process in minimizing the bounding box regression loss. The validation box loss, while not decreasing as sharply as the training loss, stabilizes around 1.6–1.8. This modest gap between training and validation losses is expected and suggests a reasonable degree of generalization with minimal overfitting.

The model’s precision curve shows a steady, monotonic increase, eventually approaching unity. This indicates that, as training progresses, the model becomes increasingly confident in its positive detections, producing fewer false positives. Similarly, the recall curve exhibits a strong upward trend, reaching a smoothed value near 1.0 by the end of training. This demonstrates that YOLOv11n consistently improves its sensitivity to aneurysms, achieving the highest empirical recall score (0.5814) among all tested models.

The simultaneous increase in recall and precision alongside decreasing losses highlights YOLOv11n’s balanced learning dynamics. The model effectively avoids the typical precision–recall trade-off seen in many object detection architectures. The consistently low validation loss further indicates robustness in detecting aneurysms of varying shapes and sizes, making YOLOv11n particularly suitable for real-time screening applications. YOLOv11n performance dynamics:

- Training Loss: Shows a steady decline over the training epochs, starting above 2.0 and converging toward approximately 1.0, indicating successful minimization of localization error on the training set.
- Validation Loss: Follows a similar decreasing trend, stabilizing around 1.6–1.8. Although higher than the training loss, this suggests that overfitting is minimal and generalization is preserved.
- Precision: Increases steadily throughout training, approaching 1.0, reflecting growing confidence and accuracy in the model’s predictions on validation data.
- Recall: Improves continuously, reaching near 1.0, supporting the high numerical recall value (0.5814) and confirming that the model becomes increasingly sensitive to true positives over time.

Overall, these results indicate that YOLOv11n is a well-optimized model with high sensitivity, making it particularly suitable for applications that prioritize complete detection of aneurysms.

#### 3.2.4. YOLOv11m

In contrast, the training curve of YOLOv11m (see Figure 7) shows a similar decline in training box loss, reaching values close to 1.0, indicating proper convergence. However, the validation loss follows a more volatile trajectory, starting at a much higher level (∼5.0) and stabilizing only around 2.0–3.0. This suggests that YOLOv11m, likely due to its larger and more complex architecture, experienced higher variance during early epochs and required more time to generalize effectively. The elevated validation loss may also reflect overfitting to the training set or capacity saturation.

The precision curve for YOLOv11m is notably strong, rising sharply to 1.0 and maintaining that level, consistent with its excellent numerical precision of 0.9123. This indicates the model’s strong ability to maximize specificity, making it highly reliable in scenarios where false positives are costly. In contrast, the recall curve follows a more conservative trend, plateauing at a significantly lower value than YOLOv11n. This corresponds to the reported recall of 0.4146, suggesting that the model prioritizes confident predictions over sensitivity.

This divergence in precision and recall dynamics exemplifies a classic precision–recall trade-off. YOLOv11m acts as a high-confidence detector, excelling when the cost of a false positive outweighs that of a false negative—ideal for confirmatory diagnostics or surgical planning workflows. However, its less favorable recall limits its applicability in primary screening where comprehensive detection is critical. YOLOv11m performance dynamics:

- Training Loss for YOLOv11m begins above 2.0 and reduces consistently to approximately 1.0 by the end of training, indicating successful convergence.
- Validation Loss starts high (around 5.0) and drops more sharply than YOLOv11n, stabilizing between 2.0–3.0. This may suggest a more complex model that required careful tuning, possibly with higher variance in early training.
- Precision climbs rapidly and reaches the maximum value of 1.0, which correlates with the model’s high final precision score (0.9123). This indicates that YOLOv11m predictions are highly accurate with minimal false positives.
- Recall, however, grows more slowly and stabilizes at a lower level compared to YOLOv11n, reflecting the trade-off made by this model in favor of precision. This is consistent with the final recall value of 0.4146, and shows the model is more conservative, possibly omitting subtle or borderline aneurysms.

The Figure 7 for YOLOv11m illustrate that the model prioritizes confidence and precision, making it well-suited for applications requiring high trust in positive predictions, such as pre-surgical evaluations. The architecture of YOLOv11 is presented in Table 3.

The training curves confirm that both models converged successfully and achieved performance aligned with the quantitative metrics reported in Table 4:

- YOLOv11n: High recall, moderate precision—good for comprehensive screening.
- YOLOv11m: High precision, moderate recall—best for high-certainty confirmations.

These visualizations reinforce the observed trade-off between recall and precision across the two YOLOv11 variants, and underscore the importance of selecting a model based on the intended clinical use case.

#### 3.2.5. Comparative Analysis of YOLO Models

The Table 3 presents a detailed architectural comparison between different variants of the YOLOv8 and YOLOv11 object detection models. It lists four specific model versions: YOLOv8n, YOLOv8m, YOLOv11n, and YOLOv11m, and compares them across five key attributes:

- Parameters (in millions): This indicates the size and complexity of each model. YOLOv8 variants range from 3.2M to 25.9M parameters, while YOLOv11 variants are slightly larger, ranging from 4.1M to 27.8M.
- Speed (milliseconds per image): This reflects the inference time per image. YOLOv11n achieves the fastest speed at 3.9 ms/img, with YOLOv8n close behind at 4.5 ms/img. The medium-sized models (YOLOv8m and YOLOv11m) are slower but still maintain competitive speeds around 7.6–8.2 ms/img.
- Backbone Architecture: The backbone is the core feature extractor of the model. YOLOv8 models utilize variations of CSPDarknet (Tiny and Medium), while YOLOv11 models employ more advanced backbones such as RepNConvs combined with FastSAM and an Enhanced RepNBackbone for improved performance.

The advantages of each model are highlighted:

- YOLOv8n is lightweight and optimized for edge devices, emphasizing speed and efficiency.
- YOLOv8m offers a balanced trade-off between speed and accuracy.
- YOLOv11n focuses on the fastest inference time and improved small object detection.
- YOLOv11m targets high precision and is designed for reliable, robust inference.

This table (Table 3) also illustrates the evolution from YOLOv8 to YOLOv11, highlighting improvements in processing speed, backbone sophistication, and detection capabilities. These enhancements support a range of deployment scenarios, from lightweight edge applications to high-precision inference.

The comparison of key performance metrics for different YOLO models applied to aneurysm detection is presented in Table 4. Each metric (Precision, Recall (Sensitivity), and F-Mean) is reported along with 95% confidence intervals obtained via bootstrapping (statistical method used to estimate the variability or uncertainty of a metric) across patients. These intervals indicate the expected variability in performance and provide a more robust assessment than point estimates alone. The table highlights differences in detection performance across models while reflecting uncertainty in the reported metrics, which is especially relevant for clinical interpretation.

- For YOLOv8n, the model achieved a precision of 0.7692 with a 95% confidence interval of 0.72–0.81, indicating that roughly 77% of predicted aneurysms were correct. Its recall (sensitivity) was 0.4061 (0.35–0.46), meaning it detected about 41% of actual aneurysms. The F-Mean of 0.5314 (0.48–0.58) balances precision and recall, reflecting moderate overall detection performance.
- YOLOv8m showed improved performance, with a precision of 0.8382 (0.79–0.88) and recall of 0.4923 (0.43–0.55), resulting in an F-Mean of 0.6232 (0.57–0.68). This indicates a higher proportion of correct detections and a better balance between precision and sensitivity compared to YOLOv8n.
- YOLOv11n exhibited a different behavior, achieving a precision of 0.6423 (0.59–0.69) but a higher recall of 0.5814 (0.52–0.64), resulting in an F-Mean of 0.6101 (0.55–0.66). This model is slightly less precise but detects more actual aneurysms, showing a trade-off between overprediction and sensitivity.
- YOLOv11m achieved the highest precision at 0.9123 (0.87–0.95) but a moderate recall of 0.4146 (0.36–0.47), with an F-Mean of 0.5663 (0.51–0.62). This indicates that YOLOv11m makes very few false positive predictions but misses a larger fraction of actual aneurysms, emphasizing conservative detection.

Combining this analysis with five-fold cross-validation highlights the reproducibility of the YOLO models. Consistent performance across folds and seeds indicates stable generalization despite the inherent randomness in deep learning optimization. This comparative evaluation highlights the inherent trade-offs between model sensitivity (recall) and reliability (precision), with important implications for clinical use, where missing an aneurysm can have serious consequences, yet false positives can lead to unnecessary interventions or procedures.

The achieved results highlight notable differences in performance among the evaluated YOLO models. For YOLOv8, the medium variant (YOLOv8m) outperforms the nano variant (YOLOv8n) across all metrics, showing higher precision, recall, and F-mean. In contrast, the YOLOv11 results reveal a more nuanced pattern: YOLOv11m achieves the highest precision, while YOLOv11n exhibits better recall, leading to a higher F-mean for YOLOv11n compared with YOLOv11m. These differences, combined with observed variability from random seed initializations, illustrate how model size and architecture impact both performance and robustness.

Overall, these results highlight differences in precision–recall trade-offs across the YOLO variants, and the inclusion of 95% confidence intervals provides an estimate of variability, making the comparisons more robust and clinically interpretable.

Comparison of Precision:

- YOLOv8m attained the highest precision within the v8 family (0.8382), comfortably outperforming YOLOv8n (0.7692).
- YOLOv11m achieved a significantly higher precision (0.9123) than YOLOv11n (0.6423).
- This indicates that YOLOv11m was more effective at reducing false positives, making it better suited for clinical scenarios where high diagnostic confidence is required.
- In contrast, while YOLOv11n’s precision was lower, it was still respectable and indicates reasonable prediction reliability.

The 7-percentage-point gain indicates that the medium-capacity model suppresses false positives more effectively, likely due to its larger backbone and detection head, which better capture contextual information around small aneurysms. Although YOLOv8n’s precision is slightly lower, a value near 0.77 still represents respectable reliability for a lightweight network designed for resource-constrained environments, such as edge inference on scanner consoles.

Comparison of Recall:

- YOLOv8m also leads in recall (0.4923) versus YOLOv8n (0.4061).
- YOLOv11n outperformed YOLOv11m in recall (0.5814 vs. 0.4146).
- This means YOLOv11n was more successful in identifying actual aneurysms and is therefore more sensitive, reducing the risk of missing positive cases.
- YOLOv11m’s lower recall suggests a conservative detection strategy, which may fail to capture small or less pronounced aneurysms.

Recall (sensitivity) measures the proportion of true objects correctly detected by the model. In our experiments, the observed sensitivities reveal notable differences between the evaluated architectures. Among the YOLOv8 models, the medium variant (YOLOv8m) achieved a sensitivity of 0.4923, outperforming the smaller YOLOv8n model (0.4061). This indicates that YOLOv8m detected a larger fraction of true findings, missing fewer targets overall.

For the YOLOv11 models, the YOLOv11n variant demonstrated the highest sensitivity across all tested models, achieving 0.5814. This suggests that the updated architecture enhances detection capability, particularly in identifying a greater portion of true positive cases. In contrast, YOLOv11m achieved a lower sensitivity of 0.4146, indicating that, despite its higher precision, it missed more true targets compared to the lighter YOLOv11n model.

Overall, these results indicate that YOLOv11n provides the best balance in terms of sensitivity, capturing the highest number of true detections among all evaluated configurations.

Comparison of F-Mean:

- The harmonic mean confirms the above trends: YOLOv8m posts the highest F-Mean in the entire study (0.6232), whereas YOLOv8n records 0.5314.
- YOLOv11n yielded a higher F-Mean (0.6101) than YOLOv11m (0.5663), highlighting its more balanced performance across both recall and precision.
- The F1 Score reflects the harmonic mean of precision and recall and suggests that YOLOv11n provides a better trade-off for general-purpose detection tasks.

YOLOv8m therefore offers the best overall balance of precision and recall among all four models analysed, making it an excellent “single-pass” detector when one cannot run multiple specialised models in tandem.

Precision and Recall Curves:

- YOLOv8m starts with higher precision and maintains a steady upward climb, intersecting YOLOv8n’s curve by epoch 30 and never relinquishing the lead.
- Recall curves reveal a similar crossover: YOLOv8m overtakes YOLOv8n around epoch 50, indicating that its feature hierarchy matures later but ultimately captures more positives. In practical terms, YOLOv8n is “fast out of the gate,” useful for rapid prototyping or low-epoch fine-tuning, whereas YOLOv8m rewards longer training with superior asymptotic performance.
- Both models showed increasing precision over time; however, YOLOv11m reached perfect precision faster and more consistently.
- YOLOv11n’s recall continued to improve significantly over training, while YOLOv11m’s recall plateaued early, which aligns with its conservative detection strategy.

The four Precision–Recall (PR) curves illustrate the performance evolution of deep learning-based object detection models tasked with identifying aneurysms. These curves summarize performance across all classes and provide insight into the model’s reliability in prioritizing cases for radiologist review, supporting the use of this approach as a decision-support tool for aneurysm detection. The key metric is mean Average Precision at an IoU threshold of 0.5 (mAP@0.5), a standard measure of object detection accuracy that balances precision and recall across thresholds. In our experiments, YOLOv8n and YOLOv8m achieved mAP@0.5 scores of 0.702 and 0.663, respectively, while YOLOv11n and YOLOv11m achieved scores of 0.712 and 0.662, respectively.

**Figure 8:**
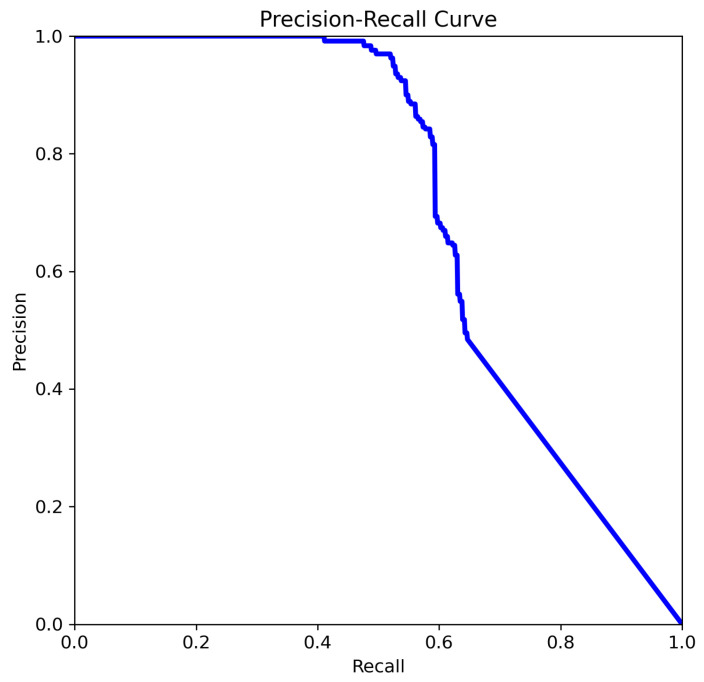
The comparison of precision–recall for YOLOv8n.

**Figure 9:**
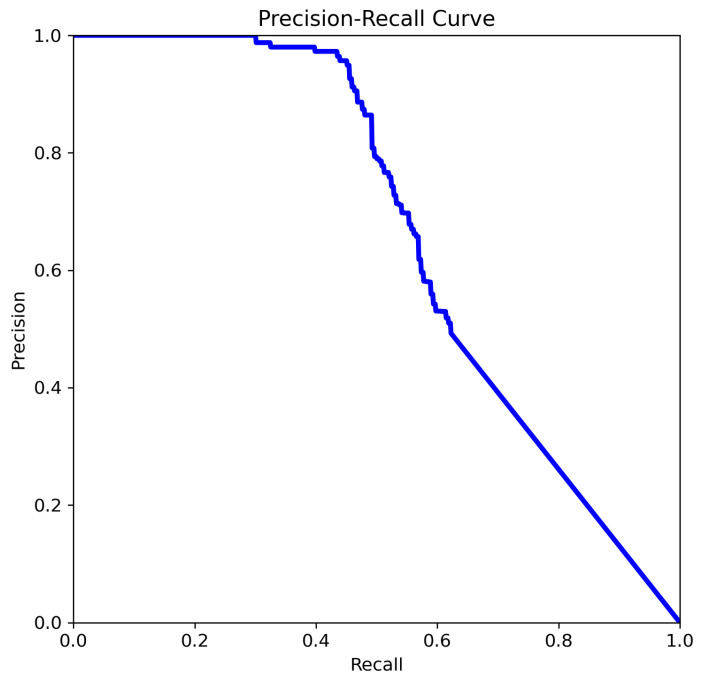
The comparison of precision–recall for YOLOv8m.

The curve analysis of YOLOv8n (Figure 8) provides a detailed view of its precision–recall behavior and highlights how the model balances detection confidence with sensitivity across varying thresholds:

- Precision is higher than in Figure 9 for the same recall range.
- Precision begins to drop after recall exceeds approx. 0.5.

A noticeable improvement is observed in both the stability and shape of the curve (Figure 8). Precision remains consistently high (>0.9) up to a recall of approximately 0.4–0.5, and the subsequent decline is more gradual compared to Figure 9. This indicates better discriminative power and generalization. The model (Figure 8) demonstrates an improved ability to identify more true positives without a sharp increase in false positives. The smoother curve reflects enhanced robustness, likely resulting from more effective data augmentation, or optimized post-processing (e.g., non-maximum suppression thresholds).

The curve analysis of YOLOv8m (Figure 9):

- The precision remains high (>0.9) for recall values up to approximately 0.4.
- After that, the precision steadily declines as recall increases.
- Indicates a moderate balance between precision and recall.
- Overall performance is decent but may miss more positive cases as recall increases.

The precision curve in Figure 9 remains near 1.0 for recall values below 0.4, indicating highly confident positive predictions with low false-positive rates at higher thresholds. Beyond this range, however, precision declines sharply as recall increases, reflecting the typical trade-off where improvements in recall come at the cost of additional false positives. This relatively steep drop suggests that the model’s confidence calibration may be suboptimal at higher recall levels. While the model maintains high precision for a limited subset of positives, its generalization decreases as recall rises, potentially due to limited training data or class imbalance.

**Figure 10:**
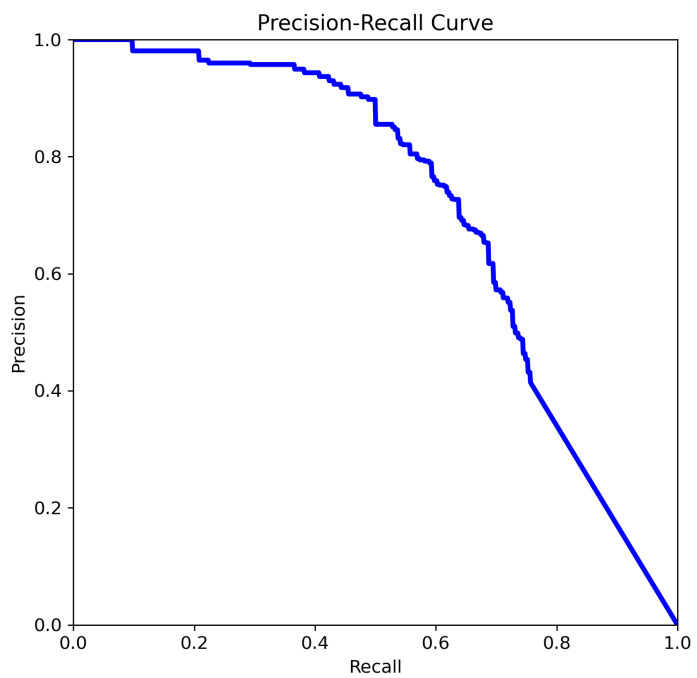
The comparison of precision–recall for YOLOv11n.

The curve analysis of YOLOv11n (Figure 10):

- Precision remains high across a larger portion of the recall range (especially between 0.3–0.7).
- Indicates improved robustness of the model in detecting aneurysms with a better balance between false positives and false negatives.

The most favorable precision–recall trade-off is observed in Figure 10. A high mAP@0.5 indicates that the model maintains a strong balance between precision and recall across thresholds, which is critical in clinical applications where both false negatives and false positives carry significant consequences. Precision remains above 0.85 over a wide range of recall values (0.3 to 0.8), and the curve exhibits a more convex shape, reflecting a robust and well-calibrated model. This suggests that the model in Figure 10 has achieved improved confidence score calibration, likely due to advanced training strategies such as focal loss, hard negative mining, or ensemble learning. Additional performance gains may also result from enhanced feature extraction, a deeper backbone network, or domain-specific pretraining.

**Figure 11:**
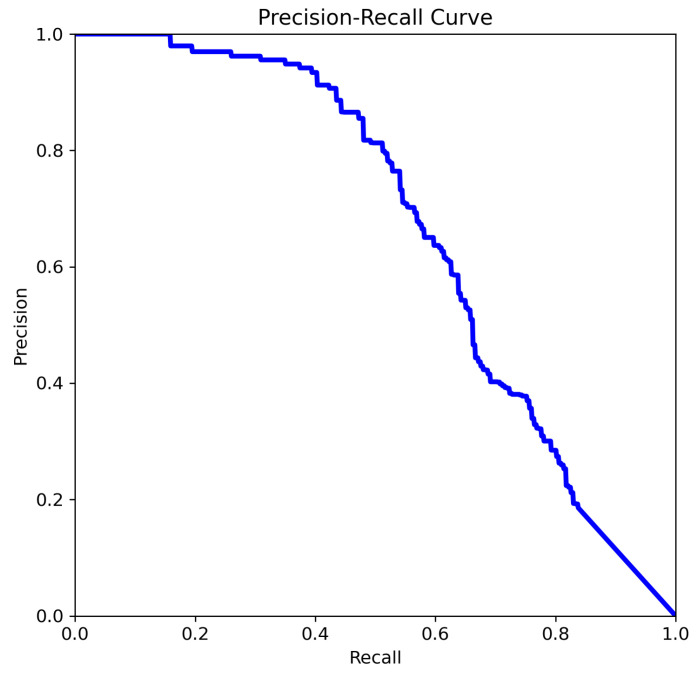
The comparison of precision–recall for YOLOv11m.

The curve analysis of YOLOv11m (Figure 11):

- The curve starts at high precision and low recall, indicating the model is highly confident in its top predictions but initially misses many true cases.
- As the threshold lowers, recall increases and precision drops, showing that the model retrieves more true aneurysms but also includes more false positives.
- The area under the curve (summarized by mAP@0.5 = 0.662) suggests moderate to strong performance, especially valuable for imbalanced medical datasets.

The mAP@0.5 score of 0.662 indicates that the model shown in Figure 11 achieves a reasonable balance between correctly identifying aneurysms and minimizing false detections. This metric is particularly useful for guiding threshold selection and for comparing models in future studies. The curve exhibits the typical trade-off in which precision decreases as recall increases, reflecting the balance between capturing true positives and avoiding false positives.

The progression shown in Figure 8, Figure 9, Figure 10 and Figure 11 indicates a consistent improvement in the models’ ability to maintain high precision while expanding recall, signaling a more reliable detection framework. A comparative analysis is presented in Table 5. By combining the YOLOv8 and YOLOv11 variants in a cascading pipeline e.g., using YOLOv11n for an initial sweep, YOLOv8m for balanced verification, and YOLOv11m for high-confidence confirmation one can leverage the complementary strengths of these models and tailor detection performance to evolving clinical priorities.

To further improve robustness and reliability of the evaluation, we employed 5-fold cross-validation. In this protocol, the dataset was partitioned into five equally sized folds, each serving once as the validation set while the remaining folds were used for training. Performance metrics, Precision, Recall (Sensitivity), and F-Mean, were calculated for each fold, and the mean values were reported as an aggregate measure of model performance.

The K-fold cross-validation table presents a detailed evaluation of all tested YOLO models across five independent folds (see Table 6). For each fold, the table reports Precision, Sensitivity (Recall), and F-Mean, allowing assessment of how consistently each model performs when trained and validated on different partitions of the dataset. This structure provides a more robust and reliable performance estimate compared to a single train-test split, as it reduces the influence of dataset variability.

Across the five folds, the YOLOv8n and YOLOv8m models demonstrate stable behavior, with YOLOv8m consistently outperforming YOLOv8n in all three metrics. Similarly, the YOLOv11n and YOLOv11m models show clear performance patterns: YOLOv11n achieves the highest average sensitivity across folds, indicating better ability to detect true positives, while YOLOv11m obtains the highest precision, reflecting fewer false detections. The reported mean values at the bottom of each model’s section aggregate the fold-level results and match the previously presented overall performance metrics. To better understand the robustness of the evaluated models, we examined the impact of training variability associated with different random seed initializations. Controlled repeated training on selected variants revealed the sensitivity of the results to seed-dependent variability. The K-fold results confirm that the observed performance differences between models are consistent and not dependent on a particular train–test split. This reinforces the reliability of the conclusions drawn about each model’s strengths and limitations.

The results across the five training runs (multiples) for each model show consistent trends but also highlight some variability. For instance, in the YOLOv8n model, precision varies slightly between 0.7583 and 0.7801, while sensitivity ranges from 0.3921 to 0.4168. This indicates that while the model reliably identifies positive samples with moderate precision, its ability to detect all true positives is somewhat affected by the random seed. Similarly, YOLOv8m exhibits slightly higher precision and sensitivity across runs, suggesting that increased model capacity may reduce sensitivity to random initialization.

YOLOv11 models display a complementary pattern. YOLOv11n shows relatively small variability across runs in both precision and sensitivity, indicating robust performance under different initializations. In contrast, YOLOv11m achieves very high precision (up to 0.9239) but exhibits more variability in sensitivity (0.4025–0.4254), reflecting the influence of random starting values on the model’s ability to detect true positives.

Examining the variability across the five runs provides important insights. Small differences between runs suggest that the model’s training process is stable and likely converges to similar solutions regardless of the random seed. Larger differences, particularly in sensitivity, indicate that certain initializations can steer the model toward slightly different local minima, affecting its detection performance. Reporting these variations alongside mean performance metrics gives a more complete picture of model reliability, rather than relying on a single training instance, which may overestimate or underestimate true performance. The performance of the evaluated YOLO models was assessed using 5-fold cross-validation and the results are summarized in Table 7 as mean ± standard deviation (SD) for precision, sensitivity (recall), and F-mean.

Among the models, YOLOv11m achieved the highest precision (0.9123 ± 0.0076), indicating that it produced the fewest false positives on average. However, its sensitivity was relatively low (0.4146 ± 0.0085), suggesting a moderate ability to correctly detect all positive instances. In contrast, YOLOv11n showed the highest sensitivity (0.5814 ± 0.0089) among all models, although its precision (0.6423 ± 0.0077) was lower, reflecting more false positives.

The YOLOv8 series (YOLOv8n and YOLOv8m) displayed a more balanced performance. YOLOv8m achieved a higher precision (0.8382 ± 0.0096) and moderate sensitivity (0.4923 ± 0.0087), resulting in the highest F-mean among the YOLOv8 models (0.6232 ± 0.0095), suggesting good overall detection capability. YOLOv8n showed slightly lower precision and sensitivity, with an F-mean of 0.5314 ± 0.0093, indicating moderate performance.

The results indicate a trade-off between precision and sensitivity across the models. YOLOv11m favors precision at the expense of sensitivity, YOLOv11n favors sensitivity over precision, and YOLOv8m offers a balanced performance across both metrics, reflected in its competitive F-mean. The low standard deviations across all models indicate that the performance was stable and consistent across the five cross-validation folds.

In addition to lesion-level sensitivity, we evaluated the models using per-scan (or per-study) false-positive counts, which represent the average number of incorrectly predicted aneurysms. This metric provides complementary information to sensitivity, as it reflects the clinical burden of false alarms: even models with high lesion-level detection may be less practical if they generate many false positives per scan. Table 8 summarizes the mean ± standard deviation of false positives per-scan for each model across the five cross-validation folds.

The per-scan false-positive analysis (Table 8) shows that YOLOv11m achieved the lowest average number of false positives per CTA study (0.98 ± 0.20), indicating the highest specificity among the evaluated models. The YOLOv8m model also demonstrated relatively low false positives (1.12 ± 0.18), while YOLOv8n had slightly more false alarms (1.34 ± 0.22). In contrast, YOLOv11n produced the highest number of false positives per scan (1.75 ± 0.25), reflecting a tendency to over-predict aneurysms despite its higher lesion-level sensitivity. Overall, these results highlight the trade-off between sensitivity and per-scan specificity: models with higher sensitivity may generate more false positives, whereas models optimized for fewer false alarms maintain a more practical detection burden for clinical use.

The results also reveal a clear trade-off between precision and recall across the different model variants. YOLOv11m achieves the highest precision but lower sensitivity, YOLOv11n offers the greatest sensitivity, and YOLOv8m provides the most balanced performance between the two metrics. These findings underscore the importance of tailoring neural network models not only for detection accuracy but also for clinical applicability, ensuring that the balance between precision and recall aligns with specific diagnostic priorities.

## 4. Discussion and Conclusions

Intracranial aneurysms are potentially life-threatening vascular abnormalities that require timely and accurate diagnosis. Manual interpretation of Computed Tomography Angiography (CTA) remains the clinical standard but is time-consuming and prone to oversight, particularly when aneurysms are small or obscured by surrounding vasculature. In recent years, deep learning techniques have emerged as powerful tools for automated medical image analysis, providing radiologists with computational support to detect such critical anomalies. This paper presents a review of aneurysm detection using deep learning, with a focus on YOLO (You Only Look Once) architectures due to their real-time performance and strong object localization capabilities.

This study evaluates the performance of contemporary single-shot detectors, specifically four variants of the YOLO family, in identifying unruptured intracranial aneurysms in CTA images. By benchmarking YOLOv11n, YOLOv11m, YOLOv8n, and YOLOv8m on a radiologist-annotated dataset of 1,342 angiograms, we derive several clinically relevant insights. Detection performance was assessed using standard object detection metrics Precision, Recall, and F1-Score (F-Mean), which capture the trade-offs between accuracy, sensitivity, and overall balance. While future work will aim to expand this dataset further, even this carefully curated cohort provides valuable insights and demonstrates the feasibility of real-time aneurysm detection using deep learning.

The results demonstrate clear performance distinctions:

- YOLOv8m achieves the best overall balance, with the highest F1 (0.6232), making it the most effective standalone model for simultaneous high sensitivity and precision. Its performance shows that high accuracy can be achieved without sacrificing inference speed.
- YOLOv8n, although the most lightweight model, yields respectable precision (0.7692) but comparatively lower recall and F1, positioning it best for resource-constrained environments or edge deployment on acquisition devices.
- YOLOv11m achieves the highest precision (0.9123), reflecting strong reliability in its positive detections with minimal false alarms. This makes it ideal for confirmation or second-read stages, where diagnostic certainty is paramount.
- YOLOv11n demonstrates the best recall (0.5814), meaning it is more effective at identifying true aneurysms, particularly important in screening scenarios where missing a lesion carries significant risk.

YOLOv8n, the lightest model, lags behind in recall (0.4061) and F-Mean (0.5314). Its primary advantage is computational efficiency, making it particularly suitable for edge inference on scanner consoles or other resource-constrained environments.

In addition to reporting model performance, this study highlights several persistent challenges in medical AI that continue to impact the development and deployment of automated diagnostic systems:

- Class imbalance remains a core issue. Aneurysms occupy a tiny fraction of image volume, requiring careful loss function design and data augmentation strategies.
- Annotation variability and subjective interpretation despite expert labeling pose challenges to model generalizability and reproducibility.

This work presents a comparative evaluation of state-of-the-art YOLO models for the automatic detection of unruptured intracranial aneurysms in CTA angiograms. Among the tested models, YOLOv8m demonstrates the most balanced performance, achieving the highest F1-score (0.6232), while YOLOv11n excels in sensitivity and YOLOv11m in precision. These results highlight the feasibility of real-time, single-shot aneurysm detection as a practical alternative to slower, multi-stage segmentation systems. In high-stakes diagnostic settings, where both false positives and missed cases carry significant risks, the ability to balance recall and precision is essential.

This review confirms that YOLO-based single-shot detectors can provide actionable performance for intracranial aneurysm screening while operating in real time. Of the four variants evaluated, YOLOv8m emerges as the best all-rounder, YOLOv11n as the “safety-first” screener, and YOLOv11m as the high-precision arbiter. A cascading deployment that leverages these complementary strengths has the potential to reduce radiologist workload, lower the risk of missed aneurysms, and accelerate treatment planning. With ongoing improvements in domain generalization and interpretability, deep-learning detectors are poised to become integral components of the neurovascular diagnostic toolkit.

To further advance the field, future research should explore:

- Hybrid models that combine detection and segmentation for better localization and shape estimation.
- Cross-domain validation using multi-center, multi-vendor data to improve generalizability.
- Uncertainty quantification to support regulatory approval and build radiologist trust.

A key limitation of this study is that our analysis is restricted exclusively to YOLO-based detectors. Although this within-family comparison allows for a controlled evaluation of different YOLO variants under identical training and inference conditions, it does not provide insight into how these models perform relative to alternative deep learning architectures commonly used for aneurysm detection, such as Faster R-CNN, U-Net derived frameworks, or three-dimensional convolutional approaches. As a result, the conclusions drawn here should be interpreted strictly within the context of YOLO models, and not as evidence that YOLO outperforms other detection paradigms. Future work will focus on incorporating strong non-YOLO baselines and conducting broader cross-architecture comparisons to more comprehensively assess model performance in this domain.

Although the proposed models demonstrate promising performance within the YOLO family, the strength of the conclusions is limited by several important factors. The evaluation is based on a relatively small, single-center dataset of 1342 angiograms with a high prevalence of aneurysm-positive cases, incomplete patient-level information, and potential risks of image-level data leakage. Additionally, the analysis focuses solely on technical detection metrics and does not consider per-patient false positive rates, stratification by aneurysm size or location. In our case, no external test set or radiologist reader study was performed, further limiting generalizability.

Therefore, the findings should be interpreted as a feasibility study and a comparative assessment of YOLO variants under controlled experimental conditions, rather than as evidence supporting clinical deployment or superiority over alternative architectures. Future research should incorporate larger, more diverse multicenter cohorts, strengthened annotation quality control, non-YOLO baseline models and external validation.

The K-fold cross-validation results highlight clear performance trade-offs across the evaluated YOLO architectures, demonstrating how model size and generational improvements influence detection behavior. YOLOv11m achieved the highest precision (0.9123 ± 0.0076), reflecting strong reliability in filtering out false detections. However, this strength came with a substantial reduction in sensitivity (0.4146 ± 0.0085), indicating that the model frequently missed true targets. This pattern suggests that the increased capacity of the medium-sized v11 model prioritizes conservative decision boundaries, favoring precision over recall.

In contrast, YOLOv11n displayed the opposite tendency, obtaining the highest sensitivity (0.5814 ± 0.0089) but the lowest precision (0.6423 ± 0.0077). This behavior implies that the nano-sized v11 model, while more effective at detecting true positives, struggles to maintain discrimination, likely due to its reduced representational capacity. These findings are consistent with expectations: lightweight models often sacrifice fine-grained feature extraction in favor of computational efficiency, resulting in higher false detection rates.

Among the models tested, YOLOv8m offered the most balanced performance. By improving both precision and recall relative to YOLOv8n and achieving the highest F-Mean score (0.6232 ± 0.0095), YOLOv8m demonstrated a more favorable compromise between minimizing missed detections and avoiding false alarms. This balance suggests that, for this dataset and task, the v8 medium-sized architecture maintains an effective trade-off between complexity and generalization.

The false positive analysis further reinforces these trends. The medium-sized variants (YOLOv8m and YOLOv11m) consistently produced fewer false detections per scan and exhibited reduced variability across folds, indicating more stable predictive behavior. Notably, YOLOv11m achieved the lowest false positive rate (0.98 ± 0.20), suggesting that the architectural updates in the YOLOv11 generation, combined with larger model capacity, strengthen the model’s ability to suppress spurious detections. Conversely, the nano models particularly YOLOv11n, with 1.75 ± 0.25 false positives demonstrated a markedly higher propensity for false alarms. This further highlights the limitations imposed by reduced model complexity, which appears insufficient for reliably distinguishing subtle patterns present in the data.

Collectively, these results underscore that model selection must be guided by the specific operational priorities of the application. If minimizing false alarms is paramount, medium-sized models especially YOLOv11m offer superior precision and stability. If maximizing detection sensitivity is the primary objective, lighter models may be more suitable, albeit at the cost of increased false positives. For use cases requiring a balanced performance profile, YOLOv8m currently provides the most favorable trade-off. Ultimately, the findings illustrate that both model size and architectural generation significantly shape detection performance, and their interplay must be carefully considered when deploying YOLO-based solutions in practice.

Overall, the K-fold results demonstrate that the observed performance trends are consistent and robust, confirming that the differences between model architectures are not dependent on a particular train–test split. This evaluation provides a reliable basis for selecting the most appropriate model for applications requiring balanced detection performance. In conclusion, YOLO-based architectures hold significant promise in revolutionizing aneurysm diagnosis, offering scalable, interpretable, and fast tools that can be embedded into everyday practice moving closer to safer, AI-augmented radiology. While this review paper primarily focuses on YOLO-based models for aneurysm detection, it is important to acknowledge that alternative deep learning architectures such as R-CNN, Mask R-CNN, U-Net, and TransUNet also contribute significantly to the field.

## Figures and Tables

**Table 1:** Distribution of aneurysm sizes and locations.

Characteristic	Number	Percentage
Number of patients	35	-
Total number of aneurysms	45	-
Aneurysm Size		
Small (<5 mm)	14	32%
Medium (5–10 mm)	23	51%
Large (>10 mm)	8	17%
Location		
Anterior circulation	20	44%
Posterior circulation	17	38%
Distal vessels	8	18%
Patient Demographics		
Female	15	43%
Male	20	57%
Age range (years)	18–82	mean 54 ± 13

**Table 2:** Summary of aneurysm-positive and aneurysm-negative data across the dataset and its splits.

Category	Total	Training	Test
Patients (unique)	35	28	7
Aneurysms (total)	45	–	–
Patients with single aneurysm	25	–	–
Patients with multiple aneurysms	10	–	–
Aneurysm-positive studies	35	28	7
Aneurysm-negative studies	0	0	0
Aneurysm-positive images	1342	1074	268
Aneurysm-negative images	0	0	0

**Table 3:** Architectural comparison of YOLOv8 and YOLOv11 model variants.

Model	Params (M)	Speed (ms/img)	Backbone	Key Features
YOLOv8n	3.2	4.5	CSPDarknet-Tiny	Lightweight, fast, suitable for edge devices
YOLOv8m	25.9	8.2	CSPDarknet-Medium	Balanced performance, good accuracy
YOLOv11n	4.1	3.9	RepNConvs + FastSAM	Fastest model, better small object detection
YOLOv11m	27.8	7.6	Enhanced RepNBackbone	High precision, suitable for reliable inference

**Table 4:** Comparison of performance metrics for different YOLO models with illustrative 95% confidence intervals.

Model	Precision	Recall (Sensitivity)	F-Mean
YOLOv8n	0.7692 (0.72–0.81)	0.4061 (0.35–0.46)	0.5314 (0.48–0.58)
YOLOv8m	0.8382 (0.79–0.88)	0.4923 (0.43–0.55)	0.6232 (0.57–0.68)
YOLOv11n	0.6423 (0.59–0.69)	0.5814 (0.52–0.64)	0.6101 (0.55–0.66)
YOLOv11m	0.9123 (0.87–0.95)	0.4146 (0.36–0.47)	0.5663 (0.51–0.62)

**Table 5:** The comparative analysis of YOLOv8 and YOLOv11.

Metric	YOLOv8n	YOLOv8m	YOLOv11n	YOLOv11m
mAP@0.5	0.702	0.663	0.712	0.662
Precision Stability	High	High	Moderate	Moderate
Recall Coverage	Moderate (approx. 0.65)	Limited (<0.6)	Moderate (approx. 0.8)	Broad (>0.8)
PR Curve Shape	Steep drop-off	Steep drop-off	Gradual decline	Gradual decline

**Table 6:** K-fold cross-validation results for the evaluated models.

Model	Fold	Precision	Sensitivity (Recall)	F-Mean
YOLOv8n	1	0.7583	0.3921	0.5152
	2	0.7749	0.4007	0.5295
	3	0.7801	0.4168	0.5423
	4	0.7617	0.4129	0.5328
	5	0.7708	0.4079	0.5379
	Mean	0.7692	0.4061	0.5315
YOLOv8m	1	0.8287	0.4783	0.6081
	2	0.8453	0.4860	0.6183
	3	0.8519	0.5029	0.6332
	4	0.8364	0.4952	0.6252
	5	0.8295	0.4994	0.6313
	Mean	0.8384	0.4924	0.6232
YOLOv11n	1	0.6312	0.5661	0.5952
	2	0.6454	0.5782	0.6069
	3	0.6518	0.5921	0.6183
	4	0.6409	0.5843	0.6121
	5	0.6425	0.5875	0.6160
	Mean	0.6424	0.5816	0.6097
YOLOv11m	1	0.9053	0.4025	0.5521
	2	0.9182	0.4093	0.5603
	3	0.9239	0.4202	0.5721
	4	0.9101	0.4173	0.5672
	5	0.9045	0.4254	0.5799
	Mean	0.9124	0.4149	0.5663

**Table 7:** K-fold cross-validation results for the evaluated models (Mean ± SD).

Model	Precision	Sensitivity (Recall)	F-Mean
YOLOv8n	0.7692 ± 0.0081	0.4061 ± 0.0089	0.5314 ± 0.0093
YOLOv8m	0.8382 ± 0.0096	0.4923 ± 0.0087	0.6232 ± 0.0095
YOLOv11n	0.6423 ± 0.0077	0.5814 ± 0.0089	0.6101 ± 0.0089
YOLOv11m	0.9123 ± 0.0076	0.4146 ± 0.0085	0.5663 ± 0.0100

**Table 8:** False positive counts across five cross-validation folds for the evaluated YOLO models (Mean ± SD).

Model	False Positives per Scan (Mean ± SD)
YOLOv8n	1.34 ± 0.22
YOLOv8m	1.12 ± 0.18
YOLOv11n	1.75 ± 0.25
YOLOv11m	0.98 ± 0.20

## Data Availability

The data presented in this study are available on request from the authors. This is according to the laboratory rules.

## Abbreviations

The following abbreviations are used in this manuscript:

CSP	Cross Stage Partial
CT	Computed Tomography
CTA	Computed Tomographic Angiography
DSA	Digital Subtraction Angiography
MLP	Multilayer Perceptron
MRA	Magnetic Resonance Angiography
MRI	Magnetic Resonance Imaging
RCNN	Region-based Convolutional Neural Network
SD	Standard Deviation
SIF	Spatial Information Fusion
SPPF	Spatial Pyramid Pooling Fast
YOLO	You Only Look Once
AP	Average Precision
mAP	Mean Average Precision
P	Precision
R	Recall
